# Continuous Hemodynamic Monitoring and Goal-Directed Therapy for Optimization in Total Knee Replacement: A Prospective Observational Study

**DOI:** 10.7759/cureus.98605

**Published:** 2025-12-06

**Authors:** Christos Christofis, Martha Kyriakou, Alexandra Arvanitaki, Konstantinos Tilkeridis, Kostas Kazakos, Theodosia Vogiatzaki, Chloropoulou Pelagia

**Affiliations:** 1 Department of Anesthesiology, University General Hospital of Alexandroupolis, Democritus University of Thrace, Nicosia, CYP; 2 Department of Health Sciences, School of Sciences, European University Cyprus, Nicosia, CYP; 3 Department of Adult Congenital Heart Disease, Royal Brompton Hospital, London, GBR; 4 2nd Cardiology Department, Aristotle University of Thessaloniki, Thessaloniki, GRC; 5 Department of Orthopaedic Surgery, University General Hospital of Alexandroupolis, Democritus University of Thrace, Alexandroupolis, GRC; 6 Department of Medicine, University General Hospital of Alexandroupolis, Democritus University of Thrace, Alexandroupolis, GRC; 7 Department of Anesthesiology, University General Hospital of Alexandroupolis, Democritus University of Thrace, Alexandroupolis, GRC

**Keywords:** clearsight system, goal-directed fluid therapy, hemodynamic monitoring, intraoperative fluid management, non-invasive monitoring, perioperative optimization, spinal anesthesia, total knee arthroplasty

## Abstract

Goal-directed fluid therapy (GDT) has become an important strategy for optimizing perioperative hemodynamics by tailoring fluid administration to each patient’s individual needs. In this prospective observational study, we evaluated the use of GDT guided by the non-invasive ClearSight monitoring system (Edwards Lifesciences Corporation, Irvine, CA, USA) in patients undergoing total knee arthroplasty (TKA) under spinal anesthesia. Forty patients were included and managed either with a GDT protocol or with standard fluid therapy. Hemodynamic parameters, fluid administration, and early postoperative outcomes were assessed. Patients in the GDT group received significantly less intraoperative crystalloid volume compared to controls and had a shorter stay in the post-anesthesia care unit. Moreover, the GDT group demonstrated more stable mean arterial pressure throughout the procedure. These findings suggest that continuous non-invasive hemodynamic monitoring can enhance intraoperative fluid optimization and improve immediate recovery following orthopedic surgery. Larger randomized trials are necessary to confirm these results and further define the clinical benefits of GDT in this setting.

## Introduction

Total knee arthroplasty (TKA) has been a cornerstone in the management of end-stage knee osteoarthritis, offering significant relief to patients suffering from debilitating pain and loss of function. Effective management of fluid administration during TKA is an integral part of perioperative care and a critical aspect of anesthesia management. Maintaining the appropriate balance in fluid levels is critical in order to optimize tissue perfusion, minimize possible complications, and promote a fast postoperative recovery. However, balancing the avoidance of hypovolemia with the prevention of hypervolemia during such a surgical procedure presents a significant challenge, requiring a delicate and precise approach [1].

Over the years, there has been a debate in the field of perioperative care and anesthesia regarding the use of traditional vs. goal-directed fluid management strategies during TKA. The traditional approach usually encompasses the use of fixed formulas or empirical estimates to guide fluid administration aiming at maintaining a stable mean blood pressure as an indirect marker of euvolemia. However, these methods lack individualization and can frequently lead to hypervolemia, resulting in perioperative cardiovascular complications, such as pulmonary edema, especially in patients with underlying cardiac disease and delayed postoperative recovery. In contrast, goal-directed fluid therapy (GDT) utilizes real-time monitoring of hemodynamic parameters to tailor fluid administration to each patient's changing hemodynamic status [2,3]. Numerous studies have shown that GDFT can reduce postoperative complications and shorten hospital stays, highlighting its potential benefits over traditional methods [4,5].

## Materials and methods

This is a prospective observational study to evaluate the impact of noninvasive hemodynamic monitoring during TKA under spinal anesthesia. The main objective was to assess hemodynamic parameters when a GDT approach was used with the ClearSight system (Edwards Lifesciences Corporation, Irvine, CA, USA) and to compare these with standard intraoperative management. The study was conducted in accordance with the Strengthening the Reporting of Observational Studies in Epidemiology (STROBE) guidelines to ensure transparency, accuracy, and completeness in reporting observational research.

Study setting and participants

The study was conducted at the 1st Department of Orthopedic Surgery, University General Hospital of Alexandroupolis, Medical School of Democritus University of Thrace. All surgeries were performed by the same orthopedic surgical team between 15 December 2017 and 20 November 2018.

Eligible participants were adult patients scheduled for elective TKA with an American Society of Anesthesiologists (ASA) score I to III [6]. Exclusion criteria included contraindications to regional anesthesia, peripheral vascular abnormalities, persistent or permanent atrial fibrillation, moderate to severe aortic regurgitation, ASA class IV or V, and refusal to provide consent. Recruitment was based on convenience sampling. A member of the research team provided verbal and written information about the study, and written informed consent was obtained prior to participation. In total, 51 patients were screened, of whom 40 were enrolled and observed either under the standard protocol or the GDT approach.

Ethical approval was granted by the Institutional Review Board of the Medical School, Democritus University of Thrace (Ref: ΕΣ:969/23-10-2017). The study adhered to the principles of the Declaration of Helsinki.

Protocols

All patients underwent a standardized preoperative assessment, which included physical examination and a brief cognitive evaluation. The same assessment was repeated postoperatively. Upon admission to the preparation ward, a peripheral intravenous catheter (18G) was placed, and the patients received 500 ml of Ringer’s lactate. Hemodynamic monitoring was initiated simultaneously using the non-invasive ClearSight system. The patients on the GDT were managed according to the GDT protocol, and the patients on the standard care group were managed according to the institutional protocol. 

**Figure 1:**
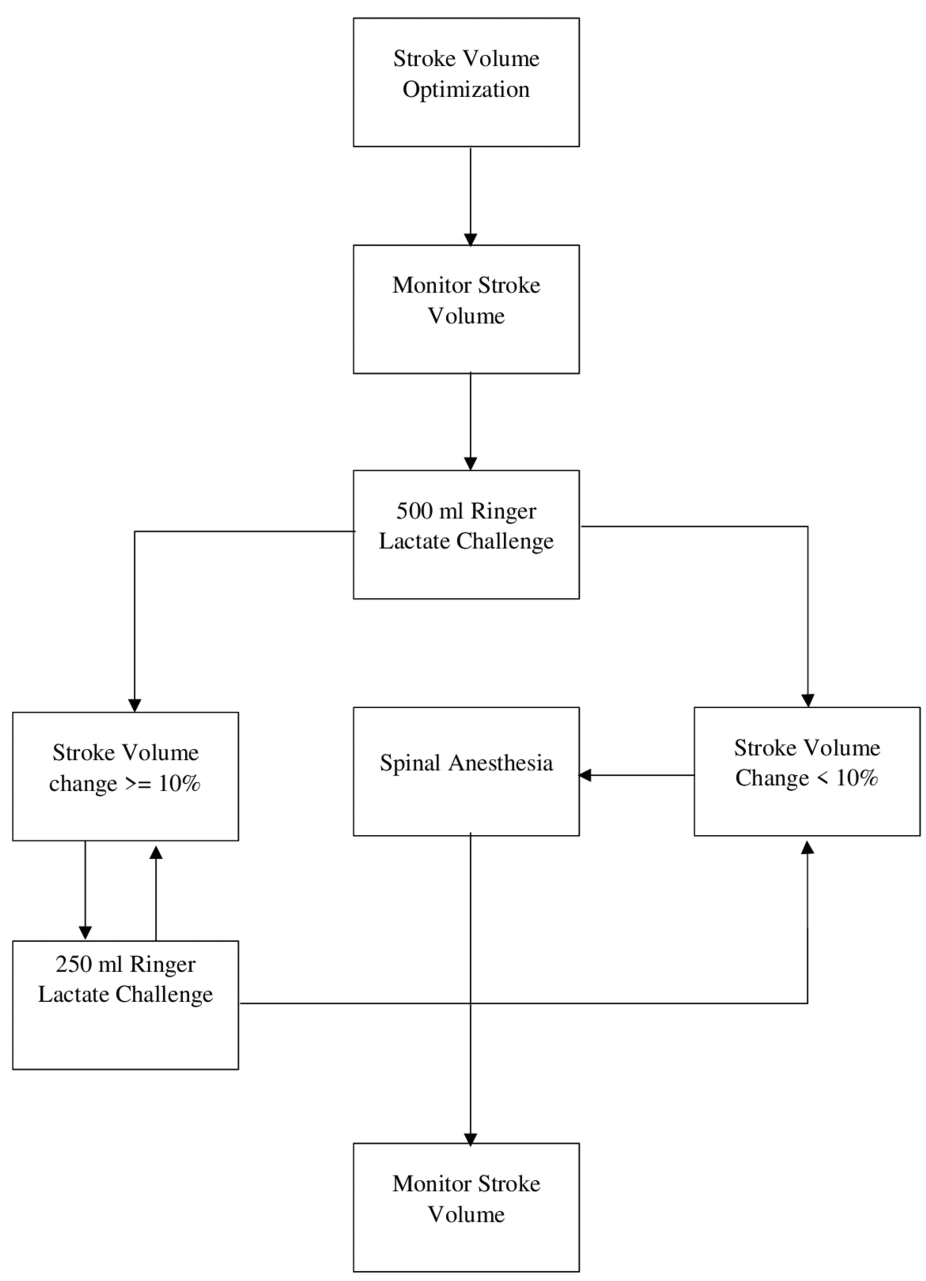
Goal-directed fluid therapy protocol. Image credits: Christofis.

In the GDT group, the algorithm demonstrated in Figure 1 was applied. An initial 500 ml Ringer's lactate solution was administered. If stroke volume (SV) increased more than 10% of the baseline value, an additional 250 ml fluid solution was administered, repeating the process as needed. After achieving the desired SV, spinal anesthesia was performed. Subsequently, fluids were administered to maintain SV up to 10% below the maximum recorded value. Vasopressors were administered when the SV value was fluid independent, but the systolic arterial pressure (SAP) (<90 mmHg) or cardiac output (CO) was low (<4 l/min). Continuous monitoring of heart rate (HR), SV, CO, SAP, diastolic arterial pressure (DAP), and mean arterial pressure (MAP) was performed in the operation theater. The defined normal ranges for these parameters are as follows: HR: 60-100 bpm, CO 4-8 l/min, SV: 60-100 ml, SAP: 90-130 mmHg, DAP: 65-90 mmHg, MAP: 60-100 mmHg.

Initially, 500 ml of Ringer's lactate solution was administered, and spinal anesthesia was performed. Hemodynamic parameters were monitored for 5 min following the completion of the bolus fluid administration to detect any significant increase in SV from the baseline value recorded upon the patient's arrival in the operating room, defined as an arbitrary SV increase of ≥10%. If a significant increase in SV was observed, the patient was classified as a fluid responder, and an additional equivalent fluid challenge was given. This process was repeated until no further increase in SV was noted, indicating fluid independence.

The EV1000 advanced monitoring platform provides a comprehensive view of hemodynamics with non-invasive ClearSight finger-cuff technology. The ClearSight system provides noninvasive access to automatically calculated, beat-to-beat hemodynamic information for a broader patient population, including patients in whom an arterial line would not typically be placed. The ClearSight system connects to the patient’s finger. It provides continuous noninvasive blood pressure (BP) from a noninvasive finger cuff in addition to key advanced hemodynamic parameters, including CO, SV, stroke volume variation (SVV), systemic vascular resistance (SVR), SAP, DAP, and MAP. The ClearSight system utilizes the volume clamp method and the physical method for continuous BP measurement and calibration. The volume clamp method maintains constant arterial volume by dynamically applying equal pressure on both sides of the arterial wall, generating a real-time finger pressure waveform. Periodic adjustments are made using a plethysmograph to track changes in smooth muscle tone, with calibration intervals starting at every 10 beats and extending to every 70 beats as stability improves. The physical method ensures accuracy through frequent recalibration, with intervals greater than 30 beats considered reliable. The system reconstructs the brachial arterial pressure waveform from the finger arterial pressure waveform using an algorithm based on extensive clinical data. SV is calculated by analyzing the systolic pressure-time integral (SPI) and employing a physiological model to determine afterload based on individual factors such as age, gender, height, and weight. CO, updated with every beat, is then derived from the product of SV and HR [7,8].

Outcomes

The primary outcome of the study was intraoperative hemodynamic stability, assessed through continuous noninvasive monitoring of MAP, CO, and SV using the ClearSight system. Fluid responsiveness, defined as an SV increase of ≥10% following a fluid challenge, was also evaluated as part of the primary outcome. The secondary outcomes included the following: total intraoperative crystalloid administration, use of vasopressors, need for blood transfusion, and duration of stay in the post-anesthesia care unit (PACU).

These outcomes were compared between the group managed according to the institutional standard protocol and the group managed under GDT.

Statistical analysis

Baseline comparisons across the two groups were explored for demographic characteristics, clinical characteristics, comorbidities, risk factors, and chronic conditions. Measures of central tendency and dispersion (mean, median, standard deviation (SD), minimum, maximum, IQR) were used for continuous variables. Additionally, the number of valid observations available for each parameter was stated. Categorical variables were presented as absolute and relative (%) frequencies. Box plots and bar plots were utilized to graphically present continuous and categorical variables, respectively. The Mann-Whitney test was used to compare the median values of a categorical variable between two independent groups. When the observations were paired, the Wilcoxon signed-rank test was used. Finally, the chi-squared (Χ²) test was used to investigate relationships between two categorical variables.

Results with a p-value <0.05 were considered statistically significant. Statistical analysis was performed with IBM SPSS v. 28 (IBM Corp. Released 2021. IBM SPSS Statistics for Windows, Version 28.0. Armonk, NY: IBM Corp) and with the RStudio program version 2023.06.0 (Posit PBC, Boston, MA, USA) [9,10].

## Results

The study sample consisted of 40 patients: 21 patients in the GDT group and 19 patients in the control group. The recruitment period lasted 12 months.

Demographic characteristics are shown in Tables 1, 2, and the clinical characteristics are presented in Table 3. There was no statistically significant difference between the two groups regarding the demographic and clinical characteristics.

**Table 1:** Categorical demographic characteristics. Statistical test: Chi-square test (p-value: 0.05). ASA: American Society of Anesthesiologists; GDT: goal-directed fluid therapy.

Demographic characteristics			N	%	Chi-square/DF	p-Value
Gender	GDT	Male	8	38.1	1.380/1	0.240
		Female	13	61.9		
	Control group	Male	4	21.1		
		Female	15	78.9		
ASA score	GDT	I	4	19.0	0.082/2	0.960
		II	12	57.1		
		III	5	23.8		
	Control group	I	4	21.1		
		II	10	52.6		
		III	5	26.3		

**Table 2:** Continuous demographic characteristics. Statistical test: Mann-Whitney (p-value: 0.05). GDT: goal-directed fluid therapy.

		N	Median	Minimum	Maximum	Mann-Whitney U	p-Value
Age (years)	GDT	21	73.0	54.0	87.0	179.5	0.587
	Control group	19	72.0	57.0	85.0		
	Overall	40	73.0	54.0	87.0		
Weight (kg)	GDT	21	86.0	64.0	115.0	167.0	0.377
	Control group	19	80.0	70.0	105.0		
	Overall	40	84.0	64.0	115.0		
Height (cm)	GDT	21	161.0	152.0	185.0	158.5	0.265
	Control group	19	160.0	153.0	175.0		
	Overall	40	161.0	152.0	185.0		
BMI (kg/m^2^)	GDT	21	31.0	26.0	36.0	181.5	0.623
	Control group	19	30.0	27.0	37.0		
	Overall	40	30.5	26.0	37.0		

**Table 3:** Clinical characteristics of the sample. Statistical test: Chi-square (p-value: 0.05). GDT: goal-directed fluid therapy.

Clinical characteristics			N	%	Chi-square/DF	p-Value
Arterial hypertension	GDT	No	7	33.3	0.014/1	0.906
		Yes	14	66.7		
	Control group	No	6	31.6		
		Yes	13	68.4		
Ischemic heart disease	GDT	No	18	85.7	0.902/1	0.342
		Yes	3	14.3		
	Control group	No	18	94.7		
		Yes	1	5.3		
Arrythmia	GDT	No	19	90.5	1.905/1	0.168
		Yes	2	9.5		
	Control group	No	19	100.0		
		Yes	0	0.0		
Respiratory disease	GDT	No	21	100.0	1.134/1	0.287
		Yes	0	0.0		
	Control group	No	18	94.7		
		Yes	1	5.3		
Diabetes mellitus	GDT	No	13	61.9	2.489/1	0.115
		Yes	8	38.1		
	Control group	No	16	84.2		
		Yes	3	15.8		
Hyperlipidemia/Hypercholesterolemia under treatment	GDT	No	13	61.9	0.351/1	0.554
		Yes	8	38.1		
	Control group	No	10	52.6		
		Yes	9	47.4		
Anxiety disorder	GDT	No	19	90.5	1.040/1	0.308
		Yes	2	9.5		
	Control group	No	15	78.9		
		Yes	4	21.1		
Smoking habits	GDT	Non-smoker	15	71.4	5.517/2	0.063
		Smoker	6	28.6		
		Former smoker	0	0.0		
	Control group	Non-smoker	16	84.2		
		Smoker	1	5.3		
		Former smoker	2	10.5		

**Figure 2:**
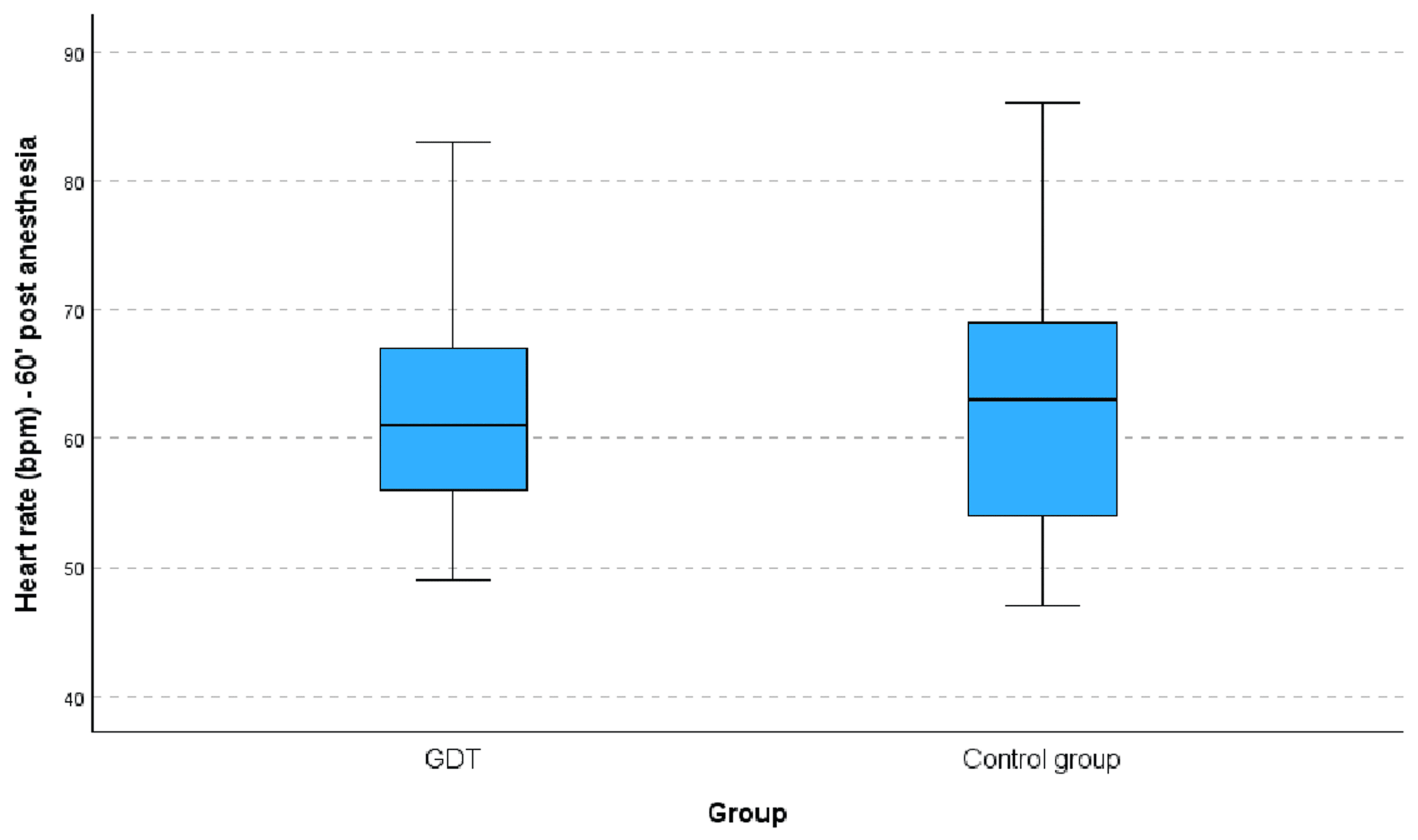
Between-group difference in HR fluctuation before and 60 min after spinal anesthesia within each group. The line inside the box represents the median, the box represents the interquartile range (25th-75th percentile), and the whiskers indicate the minimum and maximum values within 1.5 × IQR. Outliers are displayed as individual points. GDT: goal-directed fluid therapy; HR: heart rate.

HR was recorded before and 60 min after anesthesia. There was no difference between the two groups regarding HR before (p=0.098) or 60 min after anesthesia (p=0.88), although a significant fluctuation was observed during this timeframe in each group (p=0.35) (Figure 2).

**Figure 3:**
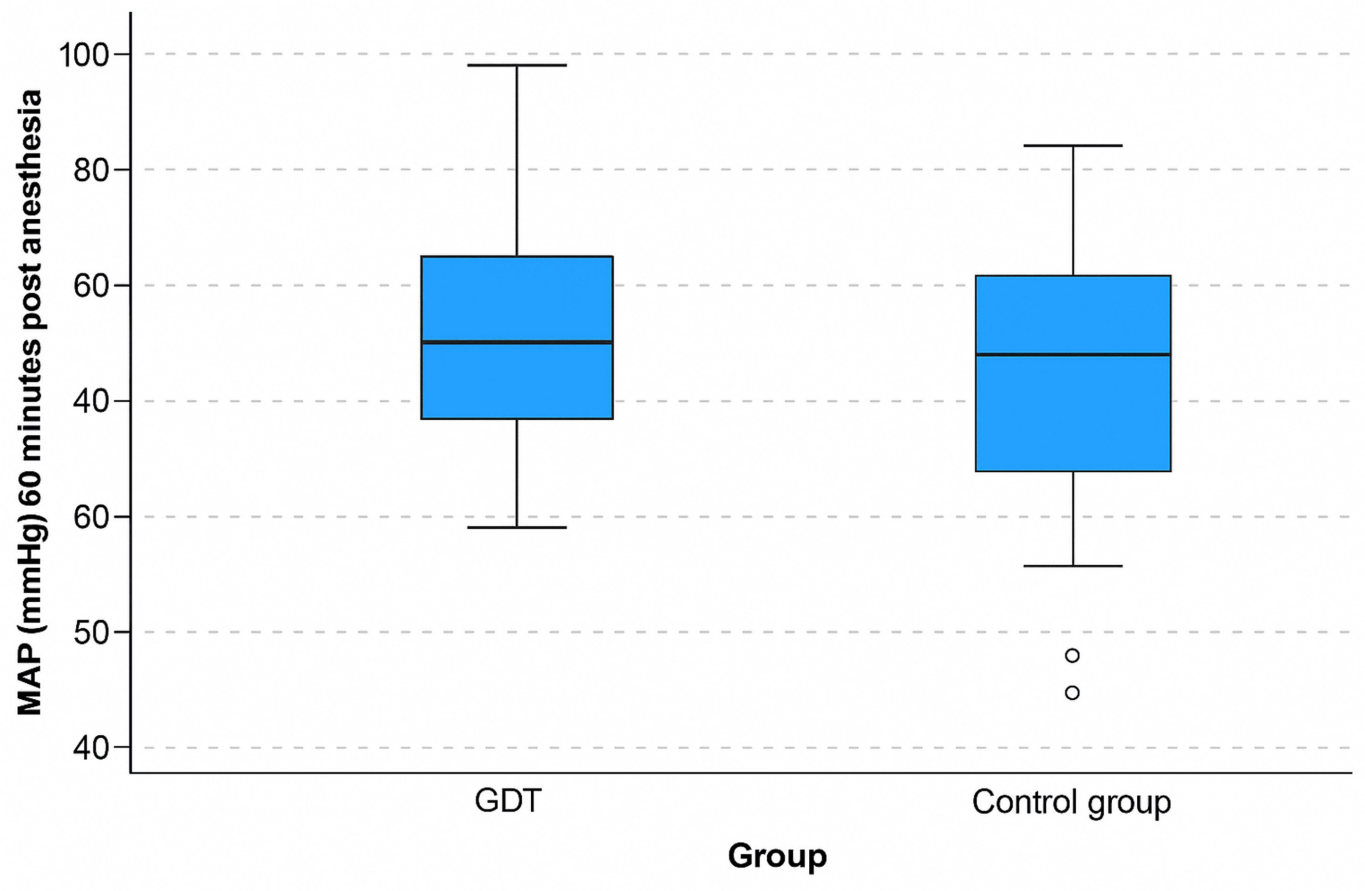
Between-group difference of MAP fluctuation within each group before anesthesia and 60 min after spinal anesthesia. The line inside the box represents the median, the box represents the interquartile range (25th-75th percentile), and the whiskers indicate the minimum and maximum values within 1.5 × IQR. Outliers are displayed as individual points. GDT: goal-directed fluid therapy; MAP: mean arterial pressure.

Regarding MAP, there was a significant difference in all three measured timepoints: 5 min (p=0.021) and 60 min after anesthesia (p<0.001), and a significant fluctuation within this time frame (p=0.007) (Figure 3).

To maintain an optimum intraoperative hemodynamic profile, crystalloids were administered according to the protocol. The median intraoperative volume of infused crystalloid was 1600 ml for the GDT group and 1800 ml for the control group (p<0.001). Lower rates of transfusion were needed for the GDT compared to the control group (p=0.17), and patients in the GDT group spent less time in the post-anesthesia care unit (PACU) (p=0.013), as shown in Table 4.

**Table 4:** Fluid administration, blood transfusion, and length of PACU stay. Statistical test: Mann-Whitney U (p-value: 0.05). PACU: post-anesthesia care unit; GDT: goal-directed fluid therapy.

Groups		N	Median	Min	Max	Mann-Whitney U	p-Value
Crystalloids (ml)	GDT	21	1600.0	1100.0	1800.0	72.0	<0.001
	Control group	19	1800.0	1100.0	2500.0		
	Total	40	1700.0	1100.0	2500.0		
Time in PACU (min)	GDT	21	15.0	15.0	25.0	113.0	0.013
	Control group	19	20.0	15.0	30.0		
	Total	40	20.0	15.0	30.0		
Number of packed blood cells transfused (units)	GDT	21	0.0	0.0	2.0	155.5	0.176
	Control group	19	1.0	0.0	2.0		
	Total	40	0.0	0.0	2.0		

## Discussion

Hemodynamic monitoring during TKA under spinal anesthesia plays a crucial role in ensuring optimal outcomes. Spinal anesthesia is often preferred due to its advantages in reducing pain, postoperative nausea, and the need for opioids, which helps facilitate faster recovery and earlier mobilization. Studies have shown that when combined with techniques like GDT, it can help maintain stable hemodynamics and improve recovery times [9].

GDT nowadays focuses on personalized hemodynamic management rather than simply maximizing CO. This approach involves assessing blood flow and fluid responsiveness to prevent both tissue hypoperfusion and hypovolemia, as well as avoiding perioperative fluid overload, all of which are linked to adverse postoperative outcomes [3]. This study demonstrates that patients monitored non-invasively with ClearSight and treated with a GDT protocol during elective primary knee replacement under spinal anesthesia showed comparable hemodynamic parameters. Following the GDT strategy, patients demonstrated more optimal MAP at all time points compared to the control group. Additionally, the investigation group exhibited a significantly less fluctuating trend in MAP. It is well established that continuous noninvasive pressure monitoring devices facilitate the rapid detection of blood pressure falls and contribute to maintaining hemodynamic stability during surgical procedures [2]. GDT facilitates the judicious use of fluids, ensuring that they are administered only when necessary and avoiding unnecessary fluid loading when hemodynamic targets are already met [4]. This strategy helps to prevent fluid overload while maintaining adequate tissue perfusion, thereby reducing the risk of postoperative complications. Additionally, when fluids alone are insufficient, the combination of vasoconstrictors to maintain MAP and inotropes to enhance SV, guided by advanced hemodynamic monitoring, can help to ensure adequate perfusion [10].

Consistent with the findings of Giglio et al. (2021) [3], patients in the GDT group received fewer fluids than the control group. This finding challenges the concern that hemodynamic optimization protocols might lead to excessive fluid administration. Instead, it supports the notion that GDT enables clinicians to deliver the appropriate amount of fluid to each patient at the right time [11-13].

Many studies have demonstrated the ability of GDT to improve postoperative outcomes and complications in patients undergoing orthopedic surgeries. In our study, a similar trend was observed concerning complications, transfusion rates, and duration of stay in the PACU. A randomized control trial would have provided a more robust impact. However, the limited data in the literature about the benefits of close hemodynamic monitoring in GDT for TKA necessitated a pilot study. Finally, despite comprehensive hemodynamic monitoring using ClearSight throughout the intraoperative period, we did not report intraoperative acute blood loss. As a result, it was not possible to correlate episodes of hypotension or reduction in SV with potential hemorrhagic events. However, blood loss was minimal, and no severe acute events were observed. All surgical procedures were conducted by the same surgical team to ensure a more reproducible technique.

This observational study, while offering valuable insights into the role of GDT during TKA under spinal anesthesia, is subject to several limitations. The non-randomized design may have introduced selection bias despite the attempt to apply consistent protocols across groups. Moreover, the relatively small sample size (n=40) limits the statistical power and the generalizability of the results to wider patient populations. In addition, the absence of data on intraoperative blood loss restricts the ability to provide a more comprehensive interpretation of hemodynamic fluctuations and the clinical relevance of SV changes.

## Conclusions

This study highlights the potential benefits of GDT guided by continuous noninvasive hemodynamic monitoring during TKA under spinal anesthesia. Patients in the GDT group exhibited more stable MAP, received lower volumes of intraoperative crystalloids, and had shorter PACU stays compared to the control group. These findings suggest that GDT can enhance perioperative hemodynamic stability and promote more efficient fluid management without compromising patient safety.

While the results are promising, the non-randomized design and small sample size limit the generalizability of the findings. Future larger-scale randomized trials are needed to confirm these outcomes and to evaluate the broader clinical and economic implications of incorporating GDT into routine orthopedic surgical care.
